# Ultrasound-Guided Intervention for Treatment of Trigeminal Neuralgia: An Updated Review of Anatomy and Techniques

**DOI:** 10.1155/2018/5480728

**Published:** 2018-04-02

**Authors:** Abdallah El-Sayed Allam, Adham Aboul Fotouh Khalil, Basma Aly Eltawab, Wei-Ting Wu, Ke-Vin Chang

**Affiliations:** ^1^Department of Physical Medicine, Rheumatology and Rehabilitation, Tanta University Hospitals, Faculty of Medicine, Tanta University, Tanta, Egypt; ^2^New Kasr El-Aini Teaching Hospital, Cairo, Egypt; ^3^Department of Radiology, Tanta University Hospitals, Tanta, Egypt; ^4^Department of Physical Medicine and Rehabilitation, National Taiwan University Hospital Bei-Hu Branch, Taipei, Taiwan

## Abstract

Orofacial myofascial pain is prevalent and most often results from entrapment of branches of the trigeminal nerves. It is challenging to inject branches of the trigeminal nerve, a large portion of which are shielded by the facial bones. Bony landmarks of the cranium serve as important guides for palpation-guided injections and can be delineated using ultrasound. Ultrasound also provides real-time images of the adjacent muscles and accompanying arteries and can be used to guide the needle to the target region. Most importantly, ultrasound guidance significantly reduces the risk of collateral injury to vital neurovascular structures. In this review, we aimed to summarize the regional anatomy and ultrasound-guided injection techniques for the trigeminal nerve and its branches, including the supraorbital, infraorbital, mental, auriculotemporal, maxillary, and mandibular nerves.

## 1. Introduction

A common cause of chronic facial pain syndrome is trigeminal neuralgia, which can be alleviated by injecting the superficial branches of the nerve, such as the supraorbital, infraorbital, and mental nerves, and deep injection of the maxillary nerve in the pterygopalatine fossa and/or the mandibular nerve posterior to the lateral pterygoid plate [1]. Isolated entrapment of the abovementioned nerves is not rare, but treatments using palpation guidance can be challenging because substantial portions of the nerves lie underneath the skull bone. The use of high-resolution ultrasound facilitates real-time visualization of peripheral nerves and adjacent soft tissue structures, such as tendons, ligaments, muscles, vessels, and subcutaneous fat [2]. Ultrasound-guided intervention allows precise targeting of the affected nerves without collateral damage to the nearby vessels and prevents accidental nerve injury, vascular thrombosis, and postinjection hematoma [3–6]. In this review, we aimed to summarize the regional anatomy and ultrasound-guided injection techniques for the commonly affected branches of the trigeminal nerve, including the supraorbital, infraorbital, mental, auriculotemporal, maxillary, and mandibular nerves.

## 2. Technical Considerations and Regimen for Treatments

All of the sonographic images presented in this review were obtained using MyLab 5 (Esaote Europe B.V., Maastricht, Netherlands). A 10–18 MHz high-frequency linear transducer was used to scan superficial structures. To image deeper structures, such as the lateral pterygoid muscle and plate, a 1–5 MHz curvilinear transducer was used. During the power Doppler examination, the Doppler frequency was set to 6.6 MHz.

To perform the superficial nerve block, 1 to 3 ml of local anesthetic, for example, 0.5% lidocaine, can be injected using a 25-gauge 1.5-inch needle. For deeper nerve blocks, 3 to 5 ml of the anesthetic can be injected using a 22-gauge 3-inch spinal needle. Potential complications include bleeding, hematoma, infection, and hypersensitivity reaction to the injectate. For longer pain relief, the deep injection can be performed using glycerol (100%), alcohol (50–70%), or phenol (5–10%). Because of the serious complications of the abovementioned neurolytic agents, such as permanent sensory deficit, severe allergic reactions, and tissue necrosis, they are gradually replaced by safer and more effective treatments like radiofrequency or cryoablation which may be considered for recalcitrant cases [7, 8].

## 3. Supraorbital Nerve

### 3.1. Anatomy

**Figure 1:**
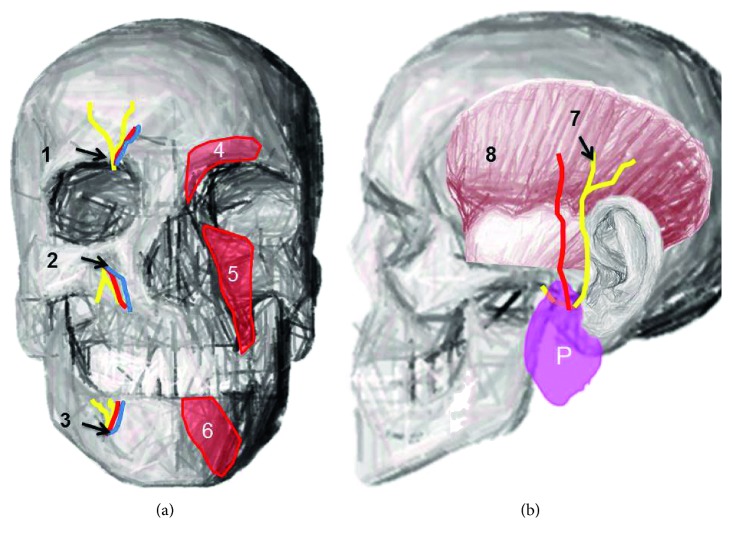
Anatomy of the supraorbital notch, infraorbital foramen, and mental foramen with corresponding neurovascular structures and the course of the auriculotemporal nerve: (a) 1 = supraorbital notch containing the supraorbital nerve and vessels; 2 = infraorbital foramen containing the infraorbital nerve and vessels; 3 = mental foramen containing the mental nerve and vessels; 4 = corrugator supercilii muscle, which is superficial to the supraorbital notch; 5 = levator labii superioris muscle, which is superficial to the infraorbital foramen; 6 = depressor labii inferioris muscle, which is superficial to the mental foramen, and (b) 7 = auriculotemporal nerve; 8 = temporalis muscle; P = parotid gland.

The frontal nerve is a branch of the ophthalmic division of the trigeminal nerve. It has two terminal branches: the larger supraorbital and smaller supratrochlear nerves. The supraorbital nerve emerges from the facial bone through the supraorbital notch which lies within the medial one-third of the supraorbital margin, 2 to 3 cm lateral to the midline (Figure 1(a) and Table 1). According to a cadaveric study, bilateral supraorbital notches were present in 49.07% of skulls, bilateral supraorbital foramina were found in 25.93% of skulls, and a notch at one side and a foramen at the contralateral side were seen in 25% of skulls [9]. The supraorbital nerve carries sensory information from the upper eyelid, forehead, and the anterior half of the scalp, except for the area innervated by the supratrochlear nerve, which is close to the midline [10].

### 3.2. Clinical Symptoms of Nerve Entrapment

**Figure 2:**
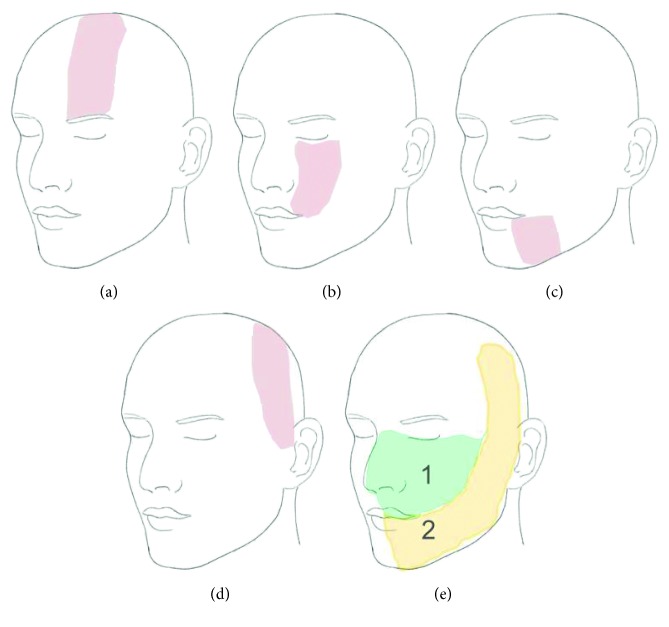
Topography of the sensory distribution of the (a) supraorbital nerve, (b) infraorbital nerve, (c) mental nerve, (d) auriculotemporal nerve, and (e) deep branches of the trigeminal nerve; 1 = area supplied by the maxillary nerve and 2 = area supplied by the mandibular nerve.

Patients with supraorbital neuralgia present with pain, tenderness, hypoesthesia, and allodynia in the territory supplied by the affected nerve. Fractures of the orbital roof, blunt trauma to the face (in boxers), tumors of the orbit, and tight swimming goggles and motorcycle helmet can cause supraorbital nerve entrapment. Imaging studies using computed tomography or magnetic resonance imaging can be used to diagnose fractures and space-occupying lesions (Figure 2(a)) [4, 11].

### 3.3. Sonoanatomy and Ultrasound-Guided Injection Technique

**Figure 3:**
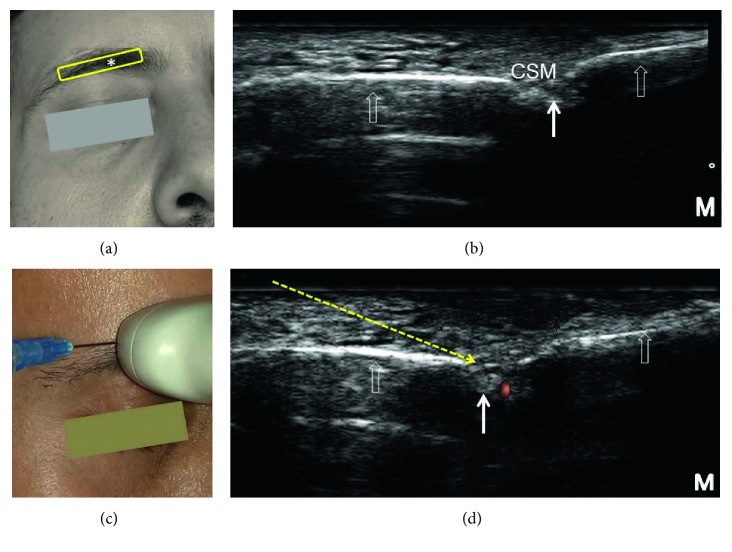
Sonoanatomy and ultrasound-guided injection technique for the supraorbital nerve: (a) the position of the transducer (yellow rectangle), (b) ultrasound imaging of the supraorbital nerve emerging from the supraorbital notch, (c) introducing the needle in the lateral-to-medial direction using the in-plane approach to target the supraorbital nerve, and (d) power Doppler image of the supraorbital vessels. The asterisk (∗) denotes the supraorbital notch on the face. The empty white arrows denote the supraorbital margin. The solid white arrow denotes the supraorbital nerve. The yellow dashed arrow denotes the needle trajectory. CSM: corrugator supercilii muscle and M: medial side. All the pictures were obtained from the face of the first author.

During this procedure, the participant lies supine with the head in the neutral position. The eye on the side of examination should be closed to prevent the coupling gel from being smeared into the eye. The transducer is placed over the medial one-third of the supraorbital margin (Figure 3(a)). The supraorbital notch can be identified as an interruption of the hyperechoic bony edge, where the supraorbital nerve and vessels exit (Figure 3(b)). For guided injection, the needle is introduced from the lateral side toward the midline using the in-plane approach to target the supraorbital nerve (Figure 3(c) and Table 1). Turning on the power Doppler mode helps with recognition of the supraorbital vessels (Figure 3(d)). The lateral edge of the transducer can be slightly lifted up to create an opening for advancement of the needle. More sterilized jelly is required to fill the space as a gel bridge (the heel-toe maneuver).

## 4. Infraorbital Nerve

### 4.1. Anatomy

The infraorbital nerve is the terminal branch of the maxillary division of the trigeminal nerve and carries sensory information from the lower eyelid, one side of the nose, and the upper lip. It emerges from the infraorbital foramen, extends to the subcutaneous layer, and is accompanied by the infraorbital vessels deep to the levator labii superioris muscle and malar fat. The infraorbital foramen lies on the anterior aspect of the maxilla bone and is approximately 1 cm below the midpoint of the infraorbital margin (Figure 1(a) and Table 1) [10].

### 4.2. Clinical Symptoms of Nerve Entrapment

The patient may complain of pain, tingling, tenderness, and allodynia in the lower eyelid, one half of the nose, and the upper lip. These symptoms may occur due to fractures of the orbital floor, malignancies of the orbit and maxilla, or blunt trauma (in boxers) which can entrap the nerve. Imaging studies using computed tomography or magnetic resonance imaging should be performed when fractures or hidden malignancies are suspected (Figure 2(b)) [6, 12].

### 4.3. Sonoanatomy and Ultrasound-Guided Injection Technique

**Figure 4:**
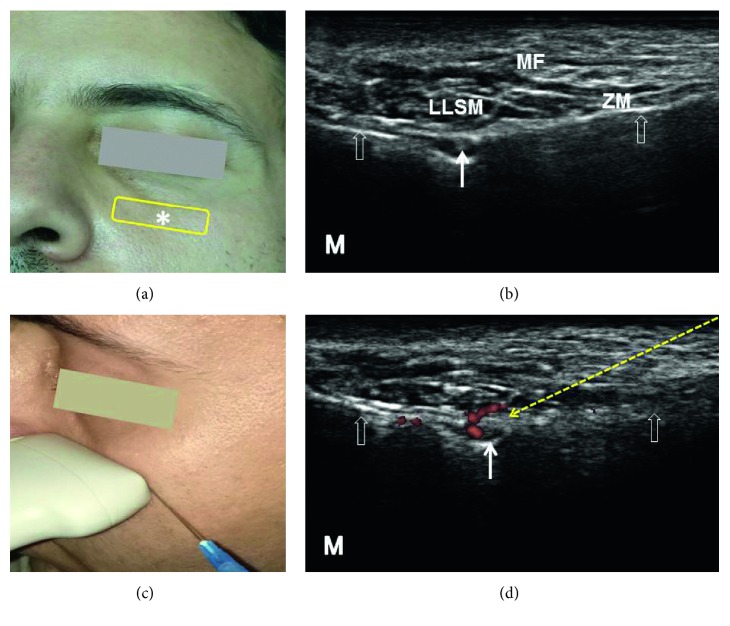
Sonoanatomy and ultrasound-guided injection technique for the infraorbital nerve: (a) the transducer position (yellow rectangle), (b) ultrasound image of the infraorbital nerve (white solid arrow) from the infraorbital foramen, (c) introducing the needle in the lateral-to-medial direction using the in-plane approach to target the infraorbital nerve, and (d) power Doppler image of the infraorbital vessels. The asterisk (∗) denotes the infraorbital foramen on the face. The empty white arrows denote the bony cortex of the maxilla. The yellow dashed arrow denotes the needle trajectory. LLSM: levator labii superioris muscle; ZM: zygomaticus minor muscle; MF: malar fat; M: medial side. All the pictures were obtained from the face of the first author.

During the procedure, the participant lies supine with the head in the neutral position. The transducer is placed over the body of the maxilla parallel to and 1 cm below the infraorbital margin (Figure 4(a)). The infraorbital foramen can be seen as an opening on the maxillary bone through which the infraorbital nerve and vessels emerge (Figure 4(b)). The needle is introduced from the lateral side toward the midline using the in-plane approach to target the infraorbital nerve in the infraorbital foramen (Figure 4(c)). The power Doppler mode should be turned on before injection to avoid damaging to the infraorbital vessels (Figure 4(d)).

## 5. Mental Nerve

### 5.1. Anatomy

The mental nerve is one of two terminal branches of the inferior alveolar nerve, which is rooted in the mandibular division of the trigeminal nerve. It supplies the skin of the chin, as well as the skin and mucous membranes of the lower lip. It emerges to the subcutaneous layer of the face through the mental foramen which lies deep to the depressor labii inferioris muscle. The foramen lies 3 cm lateral to the midline and 1 cm above the lower border of the mandible between the first and second premolar teeth (Figure 1(a) and Table 1) [13].

### 5.2. Clinical Symptoms of Nerve Entrapment

Patients usually have pain and paresthesia on the skin of the chin, as well as the skin and mucous membrane of the lower lip. The nerve can be entrapped due to fractures of the mandible, blunt trauma to the face (in boxers), dental pathologies, or malignancies of the oral cavity. The computed tomography and magnetic resonance imaging may be required for confirmation of the diagnosis of the underlying cause of entrapment (Figure 2(c)) [3].

### 5.3. Sonoanatomy and Ultrasound-Guided Injection Technique

**Figure 5:**
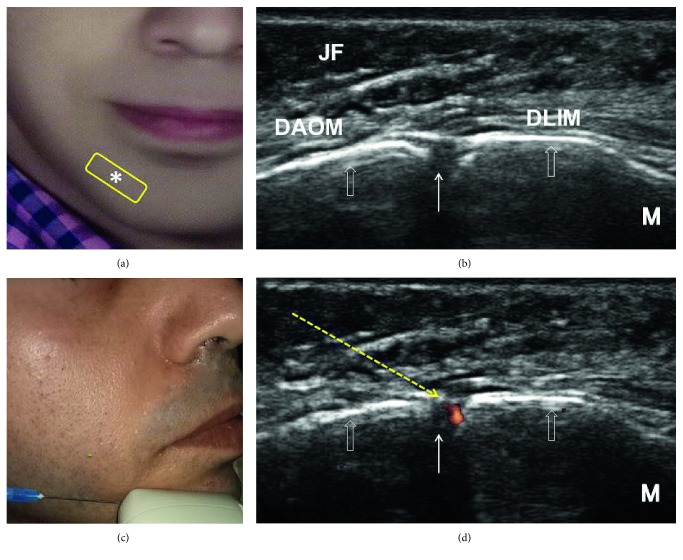
Sonoanatomy and ultrasound-guided injection technique for the mental nerve: (a) the transducer position (yellow rectangle), (b) ultrasound imaging of the mental nerve (white solid arrow), (c) introducing the needle from the lateral side toward the midline using the in-plane approach to target the mental nerve, and (d) power Doppler image used to identify the mental vessels. The empty white arrows denote the body of the mandible. The asterisk (∗) denotes the mental foramen on the face. The yellow dashed arrow denotes the needle trajectory. DLIM: depressor labii inferioris muscle; DAOM: depressor anguli oris muscle; JF: jowl fat; M: medial side. All the pictures were obtained from the face of the first author.

During the procedure, the participant lies supine with the head in the neutral position. The transducer is placed over a point located 3 cm lateral to the midline and 1 cm above and parallel to the lower border of the mandible (between the first and second premolar teeth) (Figures 5(a) and 5(b)). We can identify the mental nerve emerging from the mental foramen based on the accompanying vessel. Using the in-plane approach, the needle can be introduced from the lateral side toward the midline to target the nerve inside the mental foramen (Figures 5(c) and 5(d); Table 1).

## 6. Auriculotemporal Nerve

### 6.1. Anatomy

The auriculotemporal nerve is a branch of the mandibular division of the trigeminal nerve. It runs deep to the condylar process. The nerve courses posterior to the condylar process, pierces the parotid gland, and surfaces at the facial soft tissue. The nerve crosses over the hind part of the zygomatic arch posterior to the superficial temporal artery (Figure 1(b) and Table 1). It carries sensations from the tragus and anterior part of the ear and the posterior part of the skin over the temporalis muscle [14].

### 6.2. Clinical Symptoms of Nerve Entrapment

Patients have unilateral lancinating pain in the tragus and anterior part of the ear, as well as in the posterior part of the temporal bone (Figure 2(d)). Symptoms can be triggered by applying pressure to the area in front of the tragus. Entrapment of the nerve can occur due to tightness of the lateral pterygoid muscle secondary to temporomandibular joint dysfunction [5].

### 6.3. Sonoanatomy and Ultrasound-Guided Injection Technique

**Figure 6:**
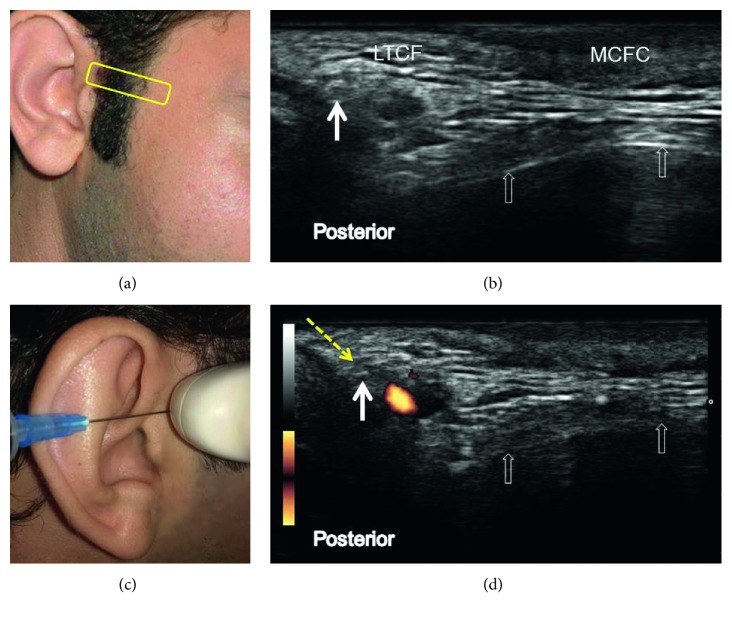
Sonoanatomy and ultrasound-guided injection technique for the auriculotemporal nerve: (a) the transducer position (yellow rectangle), (b) ultrasound imaging of the auriculotemporal nerve (white solid arrow), (c) introducing the needle in the posterior-to-anterior direction using the in-plane approach to target the auriculotemporal nerve, and (d) the power Doppler image of the superficial temporal artery. The empty white arrows denote the zygomatic arch. The yellow dashed arrow indicates the needle trajectory. MCFC: middle cheek fat compartment and LTCF: lateral temporal cheek fat. All the pictures were obtained from the face of the first author.

In this procedure, the participant lies on his or her side with the affected side of the face facing upward. The transducer is placed over and parallel to the posterior part of the zygomatic arch just above the level of the tragus (Figure 6(a) and Table 1). The auriculotemporal nerve is seen posterior to the superficial temporal artery. The needle is introduced in the posterior-to-anterior direction using the in-plane approach to target the short axis of the auriculotemporal nerve (Figures 6(b)–6(d)).

## 7. Maxillary and Mandibular Nerves

### 7.1. Anatomy

**Figure 7:**
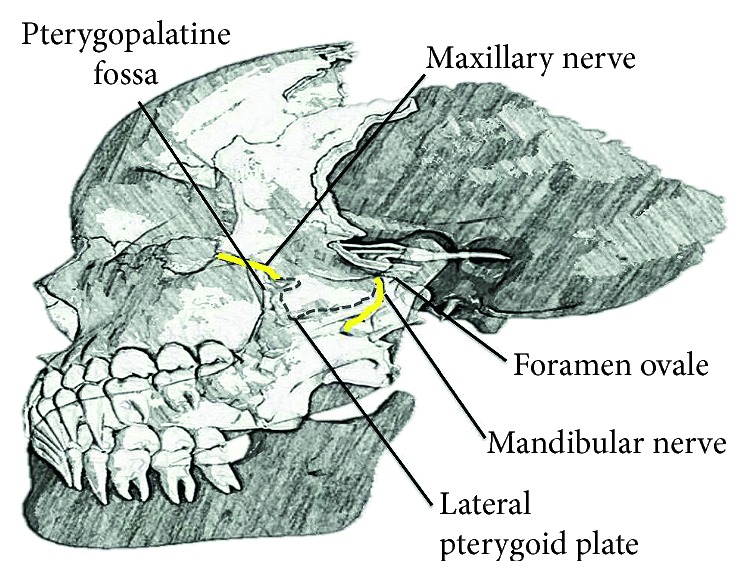
Anatomy of the maxillary and mandibular nerves related to the lateral pterygoid plate.

The Gasserian ganglion of the trigeminal nerve has 3 branches, namely, the ophthalmic and maxillary nerves, and the sensory root of the mandibular nerve. The maxillary nerve runs through the dura of the lateral wall of the cavernous sinus. It then passes through the foramen rotundum, exits the skull, and enters the pterygopalatine fossa. The maxillary nerve leaves the pterygopalatine fossa through the infraorbital fissure and becomes the infraorbital nerve in the orbital cavity (Figure 7). It carries sensations from the lower eyelid, cheek, nose, upper lip, upper teeth and gums, palate, roof of the pharynx, and the maxillary, sphenoid, and ethmoid sinuses and meninges [15]. The mandibular nerve leaves the middle cranial fossa through the foramen ovale and descends posterior to the lateral pterygoid plate (Figure 7) between the lateral and the medial pterygoid muscles. It provides motor innervation to the mylohyoid, tensor tympani, and tensor veli palatini muscles. It also carries sensory information from the anterior two-thirds of the tongue, teeth, and mucosa, as well as the periosteum of the mandible and skin of the chin and lower lip. The mandibular nerve also carries sensory information from the skin over the mandible, except for that over the mandibular angle, the tragus and anterior part of the ear, and the skin over the posterior part of the temporalis muscle up to the scalp [16].

### 7.2. Clinical Symptoms of Nerve Entrapment

Trigeminal neuralgia is usually unilateral sharp, stabbing, or burning pain that typically radiates to the area innervated by one or more divisions of the trigeminal nerve (Figure 2(e)). Pain can be triggered by irritation of the innervated skin or by activities such as eating, talking, washing the face, or cleaning the teeth. Between paroxysms, the patient is mostly asymptomatic. Imaging studies using magnetic resonance imaging and computed tomography are helpful in identifying causes, such as compression by the vessels adjacent to the nerve, mass lesions, or fractures of the skull bone [17].

### 7.3. Sonoanatomy and Ultrasound-Guided Injection Technique

**Figure 8:**
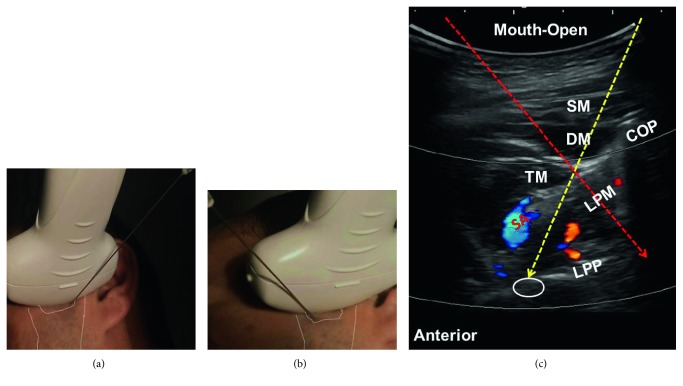
Ultrasound-guided injection technique for the maxillary and mandibular nerves: (a) the transducer position and required placement for the maxillary nerve, (b) the transducer position and required placement for the mandibular nerve, and (c) color Doppler image during mouth opening. The yellow dashed arrow represents the needle trajectory for injection of the maxillary nerve, while the red dashed arrow represents the needle trajectory for injection of the mandibular nerve. The empty white circle denotes the pterygopalatine fossa. COP: condylar process; DM: deep masseter; LPM: lateral pterygoid muscle; LPP: lateral pterygoid plate; TM: temporalis muscle; SM: superficial masseter; SA: sphenopalatine artery. All the pictures were obtained from the face of the first author.

During this procedure, the participant lies on his or her side with the affected side facing upward. Since the nerve is deeply situated, the use of a curvilinear transducer is preferred. The transducer is placed distal and parallel to the zygomatic arch to bridge the coronoid and condylar processes. The lateral pterygoid muscle can be seen originating from the condylar process and attaching to the lateral pterygoid plate. The power or color Doppler mode can be turned on to identify the sphenoid palatine artery, which is a branch of the maxillary artery, flowing to the pterygoid palatine fossa. The needle is introduced using an out-of-plane approach to target the pterygopalatine fossa (the area anterior to the lateral pterygoid plate) (Figures 8(a)–8(c); Table 1) [18, 19]. For the mandibular nerve block, the needle is introduced in the anterior-to-posterior direction to target the area posterior to the lateral pterygoid plate and between the medial and lateral pterygoid muscles (Figures 8(b) and 8(c)). Electrostimulation can be used to confirm the needle position for the deep block of the mandibular nerve. The technique of electrostimulation requires a 22 G, 10 cm insulated short beveled needle with a guard piece at 6 cm connected to a peripheral nerve simulator. The needle is inserted posterior to the lateral pterygoid plate under ultrasound guidance. The ground electrode should be placed on the anterior border of the ipsilateral masseter. The initial stimulating current should be set at 1.3 mA, with a frequency of 2 Hz. A motor response from the temporalis and masseter muscles results in a jaw jerk, and then, the current should be reduced to a threshold of 0.6 mA [20].

## 8. Conclusion

Using high-resolution ultrasound, pain interventionists can easily target the superficial branches of the trigeminal nerve and its deep branches by recognizing the adjacent muscular and bony structures. Most importantly, since the facial area is hypervascular, power Doppler imaging should be routinely turned on before intervention to avoid collateral injury to surrounding vessels. Accordingly, ultrasound-guided interventions for trigeminal neuralgia provide a safe effective solution for patients who are not responsive to or cannot tolerate oral medications and who are not appropriate candidates for surgery.

## Figures and Tables

**Table 1:** Summary of the anatomy and guided injection techniques of the trigeminal nerve and its branches.

Nerve	Bony landmark	Sensory innervation of the nerve	Accompanying vessel	Adjacent muscle	Transducer selection	Transducer placement	Needle trajectory	Ultrasound-guided technique
Supraorbital nerve	Supraorbital notch at the medial one-third of the supraorbital margin about 2 to 3 cm lateral to the midline	Upper eyelid, forehead, and the anterior half of the scalp	Supraorbital artery and vein	Corrugator supercilii muscle	Linear transducer	Medial one-third of the supraorbital margin	From lateral to medial	In-plane
Infraorbital nerve	Infraorbital foramen 1 cm below the midpoint of the infraorbital margin	Lower eyelid, half side of the nose, and the upper lip	Infraorbital artery and vein	Levator labii superioris muscle	Linear transducer	Body of the maxilla parallel to and 1 cm below the infraorbital margin	From lateral to medial	In-plane
Mental nerve	Mental foramen 3 cm lateral to the midline and 1 cm above the lower border of the mandible	Skin of the chin and lower lip and mucosa of the lower lip	Mental artery and vein	Depressor labii inferioris muscle	Linear transducer	3 cm lateral to the midline and 1 cm above and parallel to the lower border of the mandible	From lateral to medial	In-plane
Auriculotemporal nerve	Posterior zygomatic arch in front of the tragus	Anterior ear and the posterior part of the skin over the temporalis muscle	Superficial temporal artery	Temporalis muscle	Linear transducer	Parallel to the posterior part of the zygomatic arch just above the level of the tragus	From posterior to anterior	In-plane
Maxillary nerve	Pterygopalatine fossa anterior and medial to the lateral pterygoid plate	Lower eyelid, cheek, nose, upper lip, upper teeth and gums, roof of the pharynx, the sphenoid and ethmoid sinuses and meninges	Sphenopalatine artery	Lateral pterygoid muscle	Curvilinear transducer	Distal and parallel to the zygomatic arch to bridge the coronoid and the condylar processes	From posterior to anterior	Out-of-plane
Mandibular nerve	Posterior to the lateral pterygoid plate	Anterior two-thirds of the tongue, teeth, and mucosa and periosteum of the mandible, skin of the chin and the lower lip, and the skin over the mandible	Middle meningeal artery	Lateral and medial pterygoid muscles	Curvilinear transducer	Distal and parallel to the zygomatic arch to bridge the coronoid and the condylar processes	From anterior to posterior	Out-of-plane
